# Case report of entrectinib associated fulminant myocarditis

**DOI:** 10.1093/ehjcr/ytae650

**Published:** 2024-12-10

**Authors:** Gohar Rundhawa, Murtaza Ali, Ron Jacob, Edmond Obeng-Gyimah, Michael N Vranian

**Affiliations:** Department of Internal Medicine, York Hospital, WellSpan Health, 30 Monument Rd, York, PA 17403, USA; Department of Internal Medicine, York Hospital, WellSpan Health, 30 Monument Rd, York, PA 17403, USA; Department of Cardiology, York Hospital, WellSpan Health, 30 Monument Rd, York, PA 17403, USA; Department of Cardiology, York Hospital, WellSpan Health, 30 Monument Rd, York, PA 17403, USA; Department of Cardiology, York Hospital, WellSpan Health, 30 Monument Rd, York, PA 17403, USA

**Keywords:** Ventricular tachycardia, Entrectinib, Fulminant myocarditis, ECMO, Cardiotoxicity, Case report

## Abstract

**Background:**

ROS1 tyrosine kinase inhibitors are one of the primary immunotherapies for *ROS1* fusion-positive cancers. Tyrosine kinase inhibitors have markedly improved outcomes for advanced cancers previously with poor prognosis. Entrectinib is an example of an ROS1 inhibitor that can be used for lung adenocarcinoma. There are numerous adverse effects with rare cardiac side effects reported, such as heart failure and myocarditis.

**Case summary:**

A 27-year-old male being treated for lung adenocarcinoma who presented new congestive heart failure 2 weeks after starting Entrectinib. He developed refractory ventricular tachycardia, cardiogenic shock with an endomyocardial biopsy that showed active lymphohistiocytic myocarditis. With antiarrhythmic therapy, heavy sedation, mechanical circulatory support, and high-dose steroids, the patient had complete resolution of symptoms and return to baseline status.

**Discussion:**

This is a rare case with a severe complication after starting Entrectinib for lung adenocarcinoma. In the literature, this is the first case of its kind presenting with myocarditis and severe heart failure after treatment with Entrectinib. This case highlights not only using cardiac imaging, and biopsy to help guide the diagnosis, but also describe the appropriate management.

**Learning points**

- To recognize the cardiotoxicity of Entrectinib and to provide prompt and effective treatment.
- To recognize Entrectinib myocarditis can cause a type 2 reversible myocardial dysfunction.
- To understand the recommendations of obtaining electrocardiogram at baseline and periodically if at risk and left ventricular ejection fraction at baseline in patients at risk for heart failure.

## Introduction

There are several gene mutations or fusions that can be targeted in treatment of lung adenocarcinoma. *ROS1* fusions are identified in 1%–2% of non-small cell lung cancer patients and with treatment are associated with favourable outcomes. Entrecitinib, an ROS1 tyrosine kinase inhibitor (TKI), is one of the primary therapies for *ROS1* fusion-positive cancers. Tyrosine kinase inhibitors have markedly improved outcomes for advanced cancers previously with poor prognosis.^1^ The most common reported cardiac side effects include prolongation of the time of from the start Q wave to end of T wave (QT interval) and left ventricular dysfunction, however rarely pericarditis, myocardial infarction, and myocarditis have been reported.^2,3^ We report a case of Entrectinib-induced myocarditis which presented with a fulminant course requiring mechanical circulatory support and a multidisciplinary approach.

## Summary figure

**Table:** 

3 months prior to admission	Found to have deep vein thrombosis and loculated pleural effusion with new diagnosis of adenocarcinoma of the right middle lung.
2 weeks before admission	Started on Entrectinib 600 mg orally once daily.
On admission	Dyspnoea, orthopnoea, and lower extremity oedema, elevated troponin, and brain natriuretic peptide.
Hospital Day 1	Ventricular fibrillation with return of spontaneous circulation, intubated, EF 35%.
Day 2–6	On amiodarone infusion, lidocaine infusion, with heavy sedation, placed on ECMO, started on 1 g of methylprednisolone for 3 days followed by taper.
Day 6	Endomyocardial biopsy.
Day 8	Ejection fraction improved to 40%, decannulated from ECMO.
Day 11	Ejection fraction improved to 55%–60%, cardiac magnetic resonance imaging consistent with myocarditis, with eventual return to baseline status.
4 months after discharge	Patient opted to forgo chemotherapy and immunotherapy, developed malignant pericardial effusion, and passed away.

## Case report

**Figure 1:**
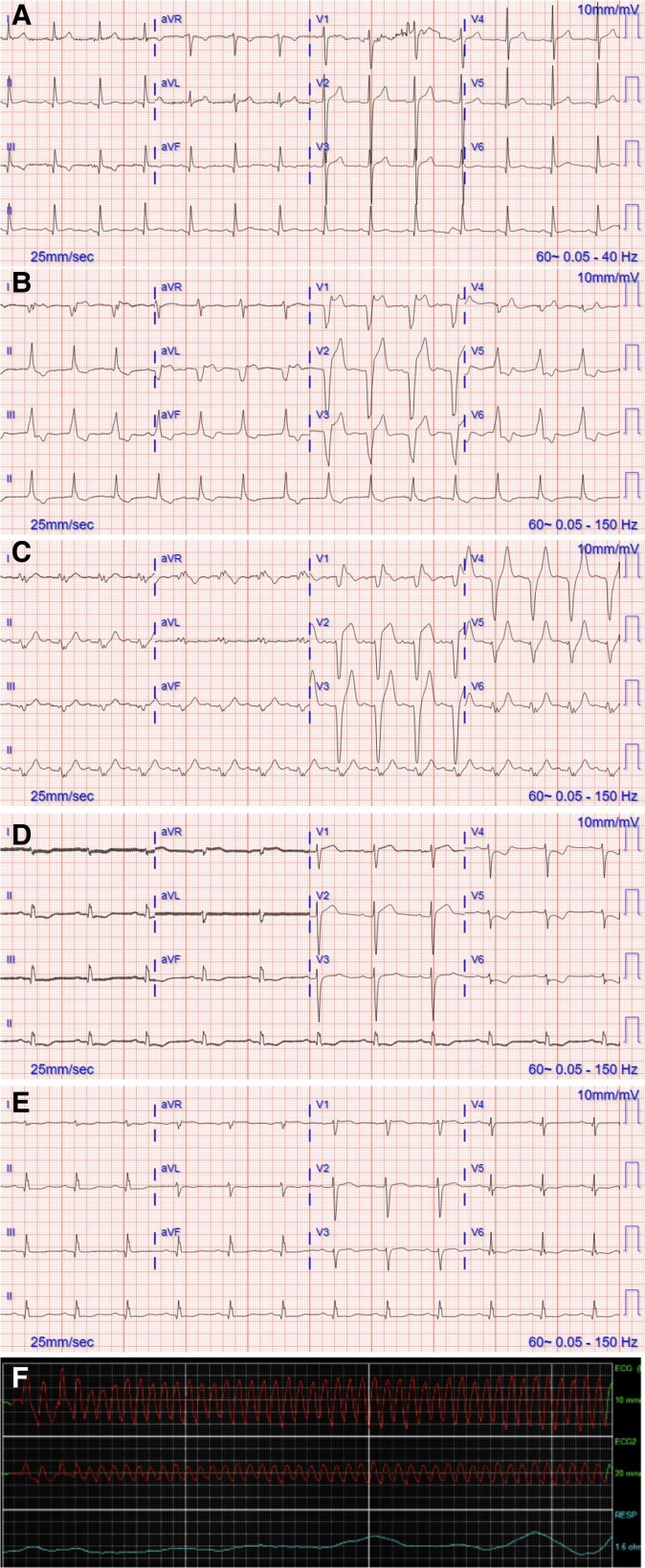
Electrocardiograms and telemetry. (*A*) Initial electrocardiogram 2 months prior to presentation. Sinus rhythm, with normal axis. Non-specific T wave changes. QTc 419 ms. (*B*) Electrocardiogram on presentation Day 1—slow ventricular tachycardia/accelerated idioventricular rhythm noted by atrioventricular dissociation, indeterminate axis, ST segment elevation secondary phenomenon to ventricular tachycardia. QTc 455 ms. (*C*) Subsequent electrocardiogram Day 1—non-specific intraventricular block, noted ST elevation as a secondary phenomenon, change in axis (extreme axis). QTc 488 ms. (*D*) Electrocardiogram Day 3—sinus rhythm with improvement in ST-T wave changes as well as QRS interval widening. Right axis deviation. QTc 414 ms. (*E*) Electrocardiogram Day 7—continued improvement in ST-T wave changes. Near normalization of axis. QTc 469 ms. (*F*) Telemetry with ventricular fibrillation.

**Figure 2:**
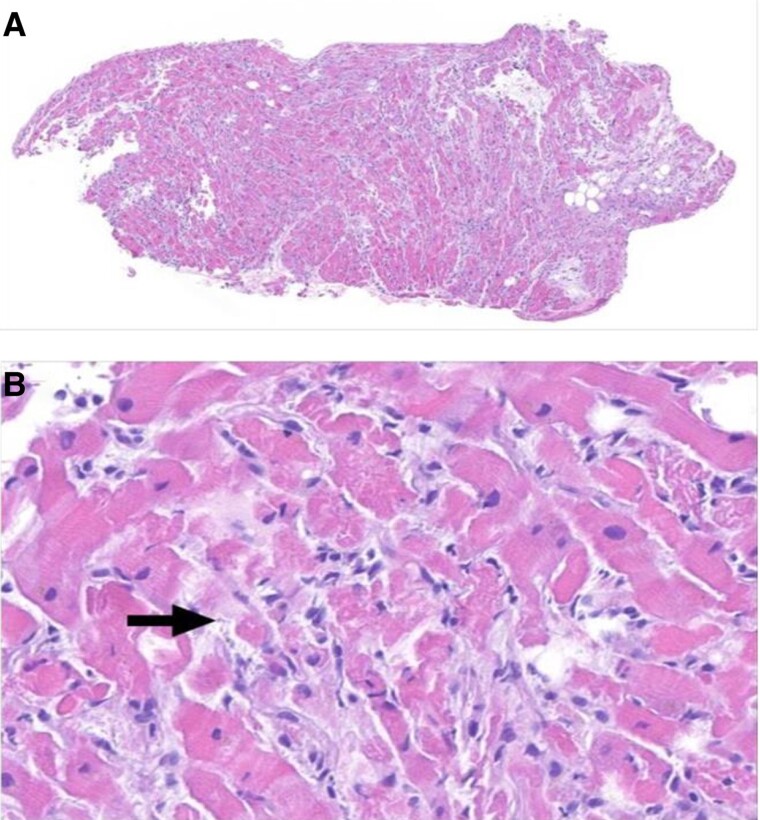
Endomyocardial biopsy. (*A*) Endomyocardial biopsy specimen shows myocardium with diffuse involvement by active lymphohistiocytic myocarditis (Haematoxylin and eosin, ×80 original magnification). (*B*) Lymphohistiocytic inflammation with associated myocyte injury (arrow), with features including hypereosinophilic damaged cells (i.e. increased staining with eosin), loss of cross-striations, and shrunken myocytes with degenerative changes (Haematoxylin and eosin, ×400 original magnification).

**Figure 3:**
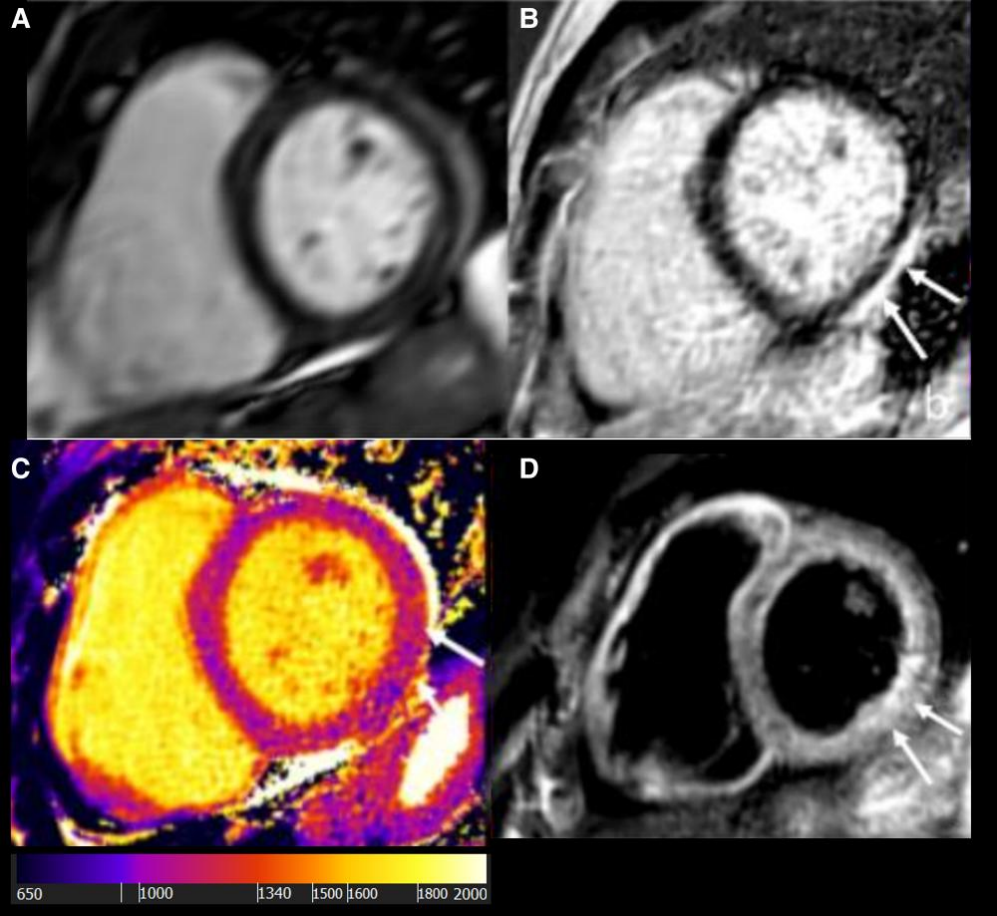
Cardiovascular magnetic resonance imaging basal short axis. (*A*) Steady-state free precession image, normal wall thickness and a small pericardial effusion. (*B*) Late Gadolinium Enhancement images with sub-epicardial enhancement in the basal inferolateral segment (arrows). (*C*) Native T1 map demonstrating severely increased T1 values in the basal inferolateral segment 1151 ms (normal 1013 ± 35 ms) (arrows). (*D*) T2 weighted black blood images demonstrating increased signal in the basal inferolateral segment suggestive of myocardial oedema (arrows).

A 27-year-old, Amish male came to medical attention with a malignant effusion found to have right lower extremity deep vein thrombosis and adenocarcinoma of the right middle lung, stage four A (IVA) (cTX, cNX, and pM1a). An *ROS1* gene fusion was identified, and he started on Entrectinib 600 mg orally once daily, 2 weeks before presentation. He presented with new onset dyspnoea and orthopnoea without any viral syndrome involving the respiratory or gastrointestinal tract. Initial vitals showed temperature of 36.2°C, blood pressure 131/77 mmHg, heart rate 88 beats per minute, respiratory rate 18 breaths per minute, and saturating 95% on room air. His physical examination was significant for bilateral lower lobe pulmonary crackles and bilateral +1 lower extremity oedema. His initial B-type naturetic peptide was 356 pg/mL. High sensitivity troponin I was >27 000 ng/L and was >27 000 ng/L on five successive checks. Initial laboratory assessment showed a lactate of 4.2 mmol/L. His aspartate aminotransferase and alanine aminotransferase were elevated 109 and 59 IU/L. Unremarkable labs included haemoglobin, white blood cell count without eosinophilia, electrolytes, creatinine, bilirubin, and alkaline phosphatase. Initial chest X-ray showed small right effusion stable from prior imaging. Computed tomography angiography imaging showed no pulmonary embolism but small right sided effusion with contrast reflux into inferior vena cava and hepatic veins with no prior imaging for comparison. A transthoracic echocardiogram (TTE) revealed an ejection fraction (EF) of 35%, without focal wall motional abnormalities. Left ventricular end-diastolic wall thickness was 1.3 cm and right ventricular diastolic mid-diameter was 5.4 cm. His left atrial and right atrial indexed volumes and left ventricular internal diastolic dimensions were normal supporting this cardiomyopathy was likely recent without chronically elevated filling pressures. He had no significant valve pathology (Supplementary material online, *Videos S1–S4*). His 12-lead electrocardiogram (ECG) showed slow ventricular tachycardia (VT) with secondary ST segment changes (*Figure 1A–E*). He was initially admitted to the general medical floors, his differential diagnosis at this point was entrectinib myocarditis, viral myocarditis, coronary artery disease, coronary spasm, and takotsubo cardiomyopathy. Left heart catheterization revealed minimal luminal irregularities without an explanation for the troponin or ECG changes. After completion of the catheterization, he had ventricular fibrillation but had return of spontaneous circulation with advanced cardiovascular life support (*Figure 1F*). He required amiodarone, lidocaine, and intubation with heavy sedation on propofol providing resolution of his ventricular arrhythmia. Hours later he had refractory VT as noted by multiple runs of sustained VT followed by pulseless VT requiring extracorporeal membrane oxygenation (ECMO) as extracorporeal cardiopulmonary resuscitation. He underwent an impella cardiac power for left ventricular venting but had subsequent ventricular ectopy prompting removal. Endomyocardial biopsy from the right ventricular septum was performed on hospital Day 6 due to concern for myocarditis with demonstrated haemodynamic instability and refractory VT. Endomyocardial biopsy showed diffuse infiltration of lymphohistiocytic inflammation involving the endomyocardium with associated myocyte injury diagnostic of active lymphohistiocytic myocarditis. No giant cells, eosinophils, or evidence of sarcoidosis were seen (*Figure 2*). His EF reached a nadir of 20% by Day 6 and started to improve by day 8%–40%. He was weaned off and decannulated from ECMO support on hospital Day 8. By hospital Day 10, he had normalized both right and left ventricular function. He underwent cardiac magnetic resonance imaging on hospital Day 11 that demonstrated a normalized EF of 60%. There was sub-epicardial delayed enhancement in the mid-inferolateral segment and severely increased T1 values in the basal inferolateral segment at 1151 ms (normal 1013 ± 35 ms). Further, a suggestion of oedema on the T2 weighted black blood images, supporting a diagnosis of myocarditis. There were no findings of prior ischaemic damage. There was a small circumferential pericardial effusion without the evidence of pericardial inflammation (*Figure 3*).

Further unremarkable labs included respiratory viral panels for Influenza A and B, coronaviruses, including COVID 19, adenovirus, *Chlamydia*  *pneumonia*, *Mycoplasma pneumoniae*, parainfluenza, enterovirus/rhinovirus, respiratory syncytial virus, *Bordetella pertussis*, and *Bordetella parapertussis*. As well as antistreptolysin O, anti-neutrophil cytoplasmic autoantibody, Coxsackie B1 and B6 antibody, HIV, cytomegalovirus, Epstein–Barr virus DNA, lyme antibody, and urine toxicology, antinuclear antibodies was 1:80 speckled. Initial sedimentation rate was 2 mm/h (normal). Initial C-reactive protein on Day 1 was normal (4.0 mg/L), but after his VT storm and progressive heart failure it rose to 35.7 (normal range <10 mg/L).

He was given 1 g of methylprednisolone on hospital Day 2 which was continued for 3 days. He subsequently was tapered to 80 mg methylprednisolone daily for 4 days, 40 mg of methylprednisolone for 2 days followed-by oral prednisone tapered over a week to 10 mg daily. He continued to have multiple morphologies of non-sustained VT and was discharged with a wearable cardiac defibrillator, amiodarone 200 mg twice per day, and metoprolol succinate 25 mg daily. Entrectinib was not continued during admission or after discharge.

He had complete resolution of symptoms with return to baseline status. A few months after discharge, a repeat TTE showed a decrease in EF to 45% without a definitive aetiology as there was no reintroduction of anti-neoplastic therapy, ischaemia, symptomatic heart failure, or arrhythmias. He had progression of his neoplastic disease subsequently causing a malignant pericardial effusion requiring pericardiocentesis with cytology showing adenocarcinoma. He and his family opted to forgo further chemotherapy or immunotherapy in favour of alternative medicine. He was then transitioned to hospice as he was not a candidate for further therapies for his cancer and passed away, 4 months after his presentation with myocarditis.

## Discussion

Tyrosine kinase not only regulates cancer cell survival and proliferation but also plays a part in cardiomyocyte survival. Inhibition will lead to myocardial dysfunction, causing cardiotoxicity including myocarditis. Our patient had a tyrosine kinase abnormality at a young age which is commonly seen in this age demographic. There are known reports of TKI causing myocarditis with only two prior reports of Entrectinib-induced myocarditis.^4^ Among 355 patients who received Entrecitinib across clinical trials 3.4% developed congestive heart failure and myocarditis in 0.3% of patients. In patients started on Entrectinib, the Food and Drug Administration recommends a baseline ECG for all the patients and TTE in those with risk for heart failure.^5^

Baseline risk assessment prior to starting a TKI includes cardiovascular history, blood pressure, lipid profile, ECG with QTc measurement. To risk stratify patients the Heart Failure Association–International Cardio-Oncology Society risk assessment tool can be used. Features associated with high risk at baseline include hypertension, cardiovascular disease, ischaemic heart disease, reduced-EF, arterial vascular disease, QTc ≥ 480 ms, age over 74 years. High-risk patients should have a cardiology consultation, TTE, and baseline B-type naturetic peptide and consideration of troponin based on drug therapy used. During treatment with a TKI each visit requires physical examination for signs of heart failure, monitoring blood pressure, lipid panel every 3–6 months, and ECG 4 weeks after initiation of therapy followed by 3 month intervals to monitor QTc and consideration for serial cardiac biomarkers and repeat TTE.^6^

Follow-up for cancer survivors requires an annual ECG and naturetic peptide. Cardiovascular follow-up is based on risk assessment after completion of therapy. High-risk adult patients should have a TTE at Years 1, 3, and 5 followed by every 5 years thereafter. Moderate risk adult patients should have TTE every 2 years. Low-risk patients should only have an annual risk assessment.^7^

Patients with myocarditis with ventricular arrhythmias are high risk and require follow-up in 1–3 months. Follow-up requires assessment of response to medical therapy, implantable cardioverter–defibrillator evaluation, and consideration for genetic testing. Maintenance of chemotherapy once cardiotoxicity occurs involves multidisciplinary discussion involving cardiology or cardio-oncology with evaluation of patient’s functional status and ability to target the known mutation. Depending on the cardiotoxicity, this may involve discontinuation of offending agent, dose reduction, or choosing an alternative agent. In our patient’s case, there was consideration to starting Crizotinib, an anaplastic lymphoma kinase inhibitor that would target the mutation; however, he had declined this therapy in favour of alternative medicine.

It is important note that several patterns of late gadolinium enhancement (LGE) are seen in myocarditis. Generally, signal increases are seen in the sub-epicardial region of the left ventricle with a variable extent through the ventricular wall. Late gadolinium enhancement may be localized to the inferolateral aspect, less commonly to anteroseptal segment and may be multi-focal or diffuse.^8^

Endomyocardial biopsy was performed on hospital Day 6 due to concern for myocarditis with demonstrated haemodynamic instability and refractory VT. The cardiac magnetic resonance imaging (MRI) was not performed prior to biopsy as the patient was on ECMO and Impella support, preventing an MRI from being obtained. As such samples were not preferentially taken from the site of LGE and were taken from the right ventricular septum. Localization of biopsy is important in the diagnosis of myocarditis and although right ventricular biopsy is technically easier with lower risk profile, myocarditis often solely involves the left ventricle. In prior studies, it has been noted that biventricular endomyocardial biopsy is diagnostic in higher percentage of cases compared with a single chamber biopsy.^9^

There are limited studies in the literature describing the use or overall efficacy of heart failure guideline-directed medical therapy (GDMT) specifically in patients with TKI use. Prior studies have suggested that using GDMT for heart failure as well as statins, contain small sample sizes, and conflicting outcomes limiting clinical significance.

There are two prior case reports of Entrectinib-related myocarditis. The first was an eosinophilic myocarditis which resolved after discontinuation of Entrectinib.^1^ The second was a non-eosinophilic myocarditis which also resolved.^10^ Our patient had neither peripheral eosinophilia nor eosinophilic infiltration in the myocardium. The time course for our patient and the prior report of non-eosinophilic myocarditis were nearly identical, starting 2 weeks after treatment initiation. Unlike prior reports, our patient had a fulminant course and had resolution of his heart failure despite no neurohormonal blockade. This supports the idea that Entrectinib myocarditis represents a type II, reversible, myocardial dysfunction. Previous case reports have also shown that rechallenge with Entrectinib did not lead to recurrent myocarditis.^1^ It is important to note that cancer therapy-related cardiac dysfunction should not be overlooked in low-risk patients given the presentation of our patient.

## Conclusion

Our finding represents the third report of myocarditis and the first of fulminant and life-threatening myocarditis associated with cardiogenic shock and VT storm which required ECMO support.^10^ It is important to recognize the cardiac adverse effects of using Entrecitinib as with prompt and effective treatment the effects of Entrectinib myocarditis can be mitigated. In our case, through rapid administration of antiarrhythmics and haemodynamic support, the patient survived VT storm and cardiogenic shock caused by fulminant myocarditis.

## Supplementary Material

ytae650_Supplementary_Data

## Data Availability

No new data were generated or analysed in support of this research.

## Lead author biography

During the year 2011, Dr Murtaza Ali studied at University Maryland, Baltimore County and obtained a bachelor’s degree in biology. He pursued medical school at American University of Antigua during the year 2015. After, he pursued his medical career as a medical resident in Internal Medicine at Wellspan York Hospital where he currently works.

## Notes

**Consent:** The patient reported in this case is deceased. Despite the best efforts of the authors, they have been unable to contact the patient’s next-of-kin to obtain consent for publication. Every effort has been made to anonymize the case. This situation has been discussed with the editors.

**Funding:** This research did not receive any specific grant from funding agencies in the public, commercial, or not-for-profit sectors.
